# Developmental and Regulatory Framework of Shoot Branching in Plants

**DOI:** 10.3390/plants15203151

**Published:** 2026-10-15

**Authors:** Linan Yue, Shuo Liu, Song Yu

**Affiliations:** 1 State Key Laboratory of Tree Genetics and Breeding, College of Forestry, Northeast Forestry University, Harbin 150040, China; 2 The Center for Basic Forestry Research, College of Forestry, Northeast Forestry University, Harbin 150040, China; 3 College of Landscape Architecture, Northeast Forestry University, Harbin 150040, China

**Keywords:** axillary meristem, branch development, plant architecture, phytohormones, transcriptional regulation, environmental signaling, molecular breeding

## Abstract

How do plants build a branch? Shoot branching is a fundamental determinant of plant architecture and is best understood as a dynamic developmental program governed by the coordinated action of multiple regulatory processes rather than by isolated pathways. Branch formation proceeds through a series of coordinated developmental transitions, from axillary meristem initiation and bud establishment to dormancy, activation, and sustained branch outgrowth, driven by interactions among phytohormones, transcription factors, environmental cues, and cellular processes. Here, we propose a developmental framework that integrates these successive stages and synthesize recent advances from both herbaceous and woody plants to illustrate how hormonal, transcriptional, and environmental signals coordinate branch development. We further highlight that these regulatory networks ultimately converge on fundamental cellular processes—including cell cycle progression, vascular differentiation, cell wall remodeling, and mechanical regulation—to execute branch formation. Finally, we discuss the evolutionary diversification of branching mechanisms and emerging technologies, including single-cell and spatial omics, comparative genomics, and artificial intelligence, that are advancing our understanding of shoot branching and accelerating the molecular design of plant architecture. This review provides an integrated framework for understanding how plants build branches and offers new perspectives for future research and crop improvement.

## 1. Introduction

Shoot branching is one of the defining features of plant architecture and represents a fundamental determinant of plant morphology, growth strategy, and ecological adaptation [1,2]. The number, position, orientation, and growth dynamics of branches collectively shape canopy architecture, influence light interception and resource allocation, and ultimately determine reproductive success, biomass accumulation, and crop yield [3,4,5]. Across the plant kingdom, branching patterns have diversified remarkably, enabling herbaceous and woody species [5] to adapt to a wide range of ecological niches and life-history strategies [6]. Consequently, understanding the mechanisms governing shoot branching has become a central objective in plant developmental biology and has attracted increasing attention in crop improvement, forest tree breeding, and molecular design of plant architecture.

Although shoot branching is traditionally described as the production of lateral branches, it is increasingly recognized as a dynamic developmental trajectory rather than a single morphogenetic event [7,8]. The formation of a functional branch involves a series of tightly coordinated transitions, including axillary meristem initiation and bud establishment to bud dormancy, bud activation, and sustained branch outgrowth [9,10]. Each transition is characterized by distinct developmental objectives, cellular behaviors, and physiological states, and must be precisely coordinated in time and space to ensure successful progression to the next stage [11,12]. Perturbation of any step may alter branch formation and ultimately reshape plant architecture. Therefore, a comprehensive understanding of shoot branching requires the identification of key regulators acting at individual stages.

Shoot branching is embedded within distinct developmental and life-history contexts in herbaceous and woody plants [5]. Although both growth forms share a broadly conserved developmental sequence encompassing axillary meristem initiation, bud establishment, dormancy or bud activation, and subsequent branch outgrowth, the temporal and physiological contexts in which these processes occur can differ substantially. Many herbaceous species establish and remodel shoot architecture within relatively short developmental cycles, whereas woody perennials repeatedly form, arrest, reactivate, and elongate axillary buds over successive seasons [5,13]. In woody plants, therefore, shoot branching is closely integrated with perennial growth, seasonal environmental cues, growth cessation, and dormancy–reactivation cycles [14]. These differences provide a biological basis for asking how a conserved branching program is reconfigured across contrasting life-history contexts [5,14]. A comparative perspective is particularly informative because it shifts the focus from identifying whether a regulatory component is present to understanding how conserved regulatory modules are deployed in different developmental contexts. Core pathways involving auxin, cytokinins, strigolactones, sugars, and transcriptional regulators control axillary meristem formation and bud outgrowth across well-characterized herbaceous model species, yet their relative contributions, temporal dynamics, and interactions with environmental and seasonal signals can vary among growth forms. Comparing herbaceous and woody plants can therefore help distinguish regulatory mechanisms that represent broadly conserved features of shoot branching from those that have been modified in association with perenniality, seasonal growth, and long-term architectural development.

Over the past decades, substantial progress has been made in elucidating the molecular mechanisms underlying shoot branching [7,9]. Genetic, physiological, and genomic studies have identified diverse regulatory pathways that govern branch development at different developmental stages [11,15]. These include phytohormone signaling networks, transcriptional regulators, environmental cues, and metabolic signals, all of which contribute to the establishment, maintenance, and outgrowth of axillary buds [7,16]. Importantly, these regulatory pathways are not independent but interact extensively through complex signaling networks to coordinate developmental transitions. Recent advances in high-throughput sequencing, single-cell transcriptomics, spatial omics, and genome editing have further expanded our understanding of the molecular landscape of shoot branching, revealing increasing regulatory complexity across both herbaceous and woody plants [17,18,19,20,21,22,23].

Despite these significant advances, current understanding of shoot branching remains largely fragmented [15]. Most studies have focused on individual regulatory pathways or specific signaling components, such as phytohormones [24,25,26,27,28], transcription factors [9,29,30,31,32,33,34,35,36,37,38], or environmental cues [15,39,40,41,42,43,44], whereas the mechanisms by which these diverse regulatory inputs are coordinated across successive developmental stages remain poorly understood. In particular, relatively little attention has been paid to how regulatory networks are dynamically integrated to control developmental transitions, or how these upstream signals ultimately converge on common cellular processes—including cell cycle progression, vascular differentiation, cell wall remodeling, and tissue mechanics—to execute branch formation [45]. Furthermore, although substantial progress has been achieved in model herbaceous species, studies in woody plants have revealed additional regulatory features associated with perennial growth and seasonal bud dynamics, yet these findings have rarely been integrated into a common developmental framework [9]. Consequently, a developmental perspective that links regulatory networks with developmental transitions and cellular execution is needed to provide a more comprehensive understanding of how plants build branches.

In this review, we propose a developmental framework that views shoot branching as a continuous developmental program encompassing axillary meristem initiation, bud establishment, dormancy, activation, and sustained branch outgrowth [8]. Based on this framework, we first summarize the developmental progression underlying branch formation and then synthesize the multilayered regulatory networks—including phytohormones, transcriptional regulation, environmental cues, metabolic signals, and cellular processes—that coordinate developmental transitions across successive stages [7]. We further compare the developmental and regulatory mechanisms operating in herbaceous and woody plants to distinguish conserved developmental principles from growth-form-specific features. Particular attention is given to the spatial and cellular contexts in which hormonal and transcriptional networks operate, as well as to how these upstream signals converge on cellular processes to drive developmental transitions and branch outgrowth. Finally, we highlight emerging technologies that are reshaping our understanding of shoot branching and accelerating the molecular design of plant architecture [46]. By integrating developmental progression, comparative biology, regulatory networks, and cellular mechanisms into a unified framework, this review aims to provide a comprehensive perspective on how plants build branches and to identify promising directions for future research, crop improvement, and tree breeding.

## 2. Developmental Framework of Shoot Branching

Shoot branching is traditionally described as the formation of lateral branches from axillary buds [9]. However, accumulating evidence from diverse plant species suggests that branch formation can be viewed as a continuous developmental process consisting of a series of partially interconnected transitions, including axillary meristem initiation, axillary bud establishment, dormancy, activation, and sustained branch outgrowth [7]. Each transition represents a distinct developmental state associated with characteristic cellular activities, physiological conditions, and regulatory requirements [11,15,16]. Although this developmental sequence is broadly conserved among many plants, the underlying regulatory mechanisms and their relative contributions may vary substantially among species and growth forms.

**Figure 1:**
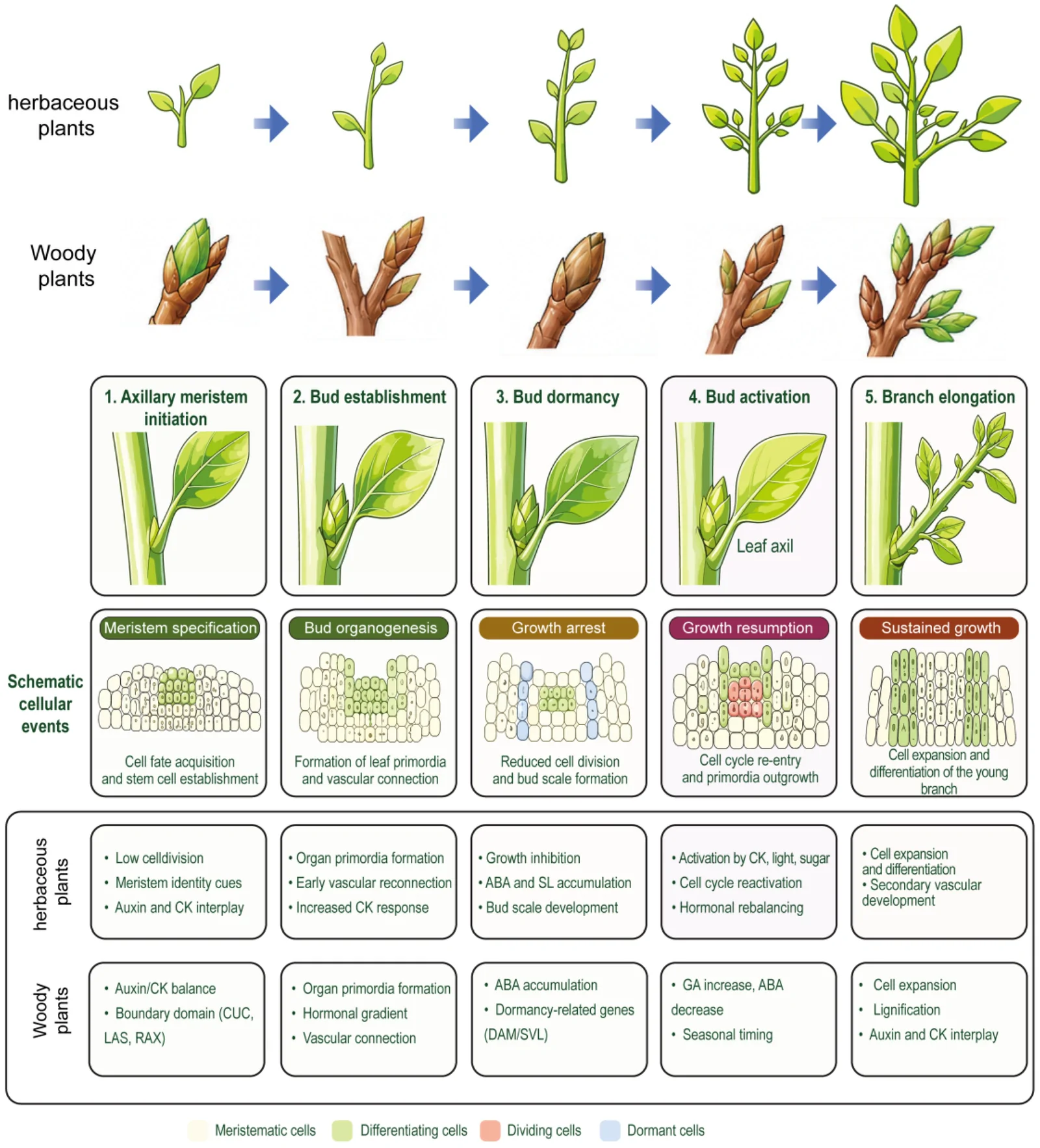
A developmental framework for shoot branching in herbaceous and woody plants. Note: Schematic representation of shoot branching as a continuous developmental program encompassing axillary meristem initiation, bud establishment, dormancy, activation, and sustained branch elongation. Both herbaceous and woody plants share this broadly conserved developmental sequence. Representative cellular events and key developmental features are shown for each stage, highlighting conserved developmental principles as well as characteristics associated with herbaceous and woody growth forms.

Although these developmental stages are often described independently, they are intrinsically linked in both time and space [7,16]. Successful progression from one stage to the next depends on the completion of preceding developmental events, while regulatory signals are continuously integrated throughout the entire developmental trajectory [47]. For example, studies in model *Arabidopsis*  *thaliana* have demonstrated that regulators involved in axillary meristem formation, such as *STM*, *CUC*, *LAS*, and *RAX*, also influence subsequent meristem maturation and bud establishment [16], In perennial woody species, including poplar, peach, and apple, seasonal hormonal changes and environmental cues have been shown to contribute to the regulation of dormancy and bud reactivation [5,15]. Likewise, the sustained elongation of lateral branches requires not only bud activation but also coordinated regulation of cell proliferation, vascular differentiation, and tissue expansion [45]. In the following sections, we summarize the developmental characteristics of each stage and discuss how their coordinated progression ultimately determines branch architecture in both herbaceous and woody plants (Figure 1).

### 2.1. Axillary Meristem Initiation

Axillary meristem (AM) initiation represents the first developmental transition in shoot branching and is considered to establish the developmental competence required for the formation of a new lateral growth axis [8]. Unlike the primary shoot apical meristem (SAM), which is specified during embryogenesis, AMs are generated post-embryonically within the leaf axil throughout the vegetative life cycle [48]. This reiterative capacity to establish new meristems contributes to the indeterminate nature of shoot branching and provides plants with remarkable developmental plasticity, enabling continuous architectural adjustment in response to endogenous developmental programs and environmental fluctuations. For many years, AM initiation was considered a localized organogenic event in which a new meristem arose de novo from differentiated leaf axil cells [49,50]. However, recent studies have fundamentally revised this view. Studies in model plants, particularly Arabidopsis, have provided evidence supporting the “detached meristem” model, proposing that a small population of meristematic cells originating from the SAM remains in the leaf axil during leaf development and subsequently gives rise to the AM [49,51,52]. These slowly dividing cells maintain low but persistent expression of *STM* and preserve meristematic competence without acquiring stem cell identity [53]. Rather than generating stem cells de novo, AM initiation in these systems therefore relies on the maintenance and subsequent reactivation of this pre-existing meristematic cell population, followed by the establishment of a functional stem cell niche [52,54,55].

The transition from meristematic competence to a functional axillary meristem is achieved through the progressive establishment of the stem cell niche and the coordinated specification of stem cell identity [52,56]. During this process, *STM* maintains undifferentiated cell fate and prevents premature differentiation, whereas *CUP-SHAPED COTYLEDON* (*CUC*) genes have been shown to contribute to establishing the boundary domain that spatially separates the emerging AM from adjacent organs [54,57,58,59]. Additional transcription factors, including *LATERAL SUPPRESSOR* (*LAS*), *REGULATOR OF AXILLARY MERISTEMS* (*RAX*), *REVOLUTA* (*REV*) and *LATERAL ORGAN FUSION* (*LOF*), collectively contribute to defining meristematic competence, organize boundary tissues, and promote stem cell establishment. Subsequently, cytokinin-mediated activation of *WUSCHEL* (*WUS*) promotes the formation and maintenance of a self-maintaining stem cell niche, allowing the newly formed AM to function as an independent meristem in well-characterized model systems [47,60,61,62]. These findings indicate AM initiation is a gradual developmental process involving both the maintenance of boundary-derived meristematic cells and the de novo establishment of stem cell identity, rather than a single developmental event [56].

Phytohormones provide the positional and temporal signals that coordinate these developmental transitions [8]. In Arabidopsis, a local auxin minimum within the leaf axil has been shown to be associated with maintain meristematic competence and establish a permissive environment for AM initiation, whereas a transient increase in cytokinin has been demonstrated to contribute to stem cell proliferation and activates WUS expression during stem cell niche formation [47]. Recent studies further suggest that auxin and cytokinin function sequentially rather than simply antagonistically, thereby coordinating the transition from boundary cells to functional stem cells. Additional regulatory inputs, including gibberellins, sugar signaling, and mechanical cues associated with boundary formation, have been proposed to modulate this process by integrating developmental and environmental information into the core meristem regulatory network [8,63].

**Figure 2:**
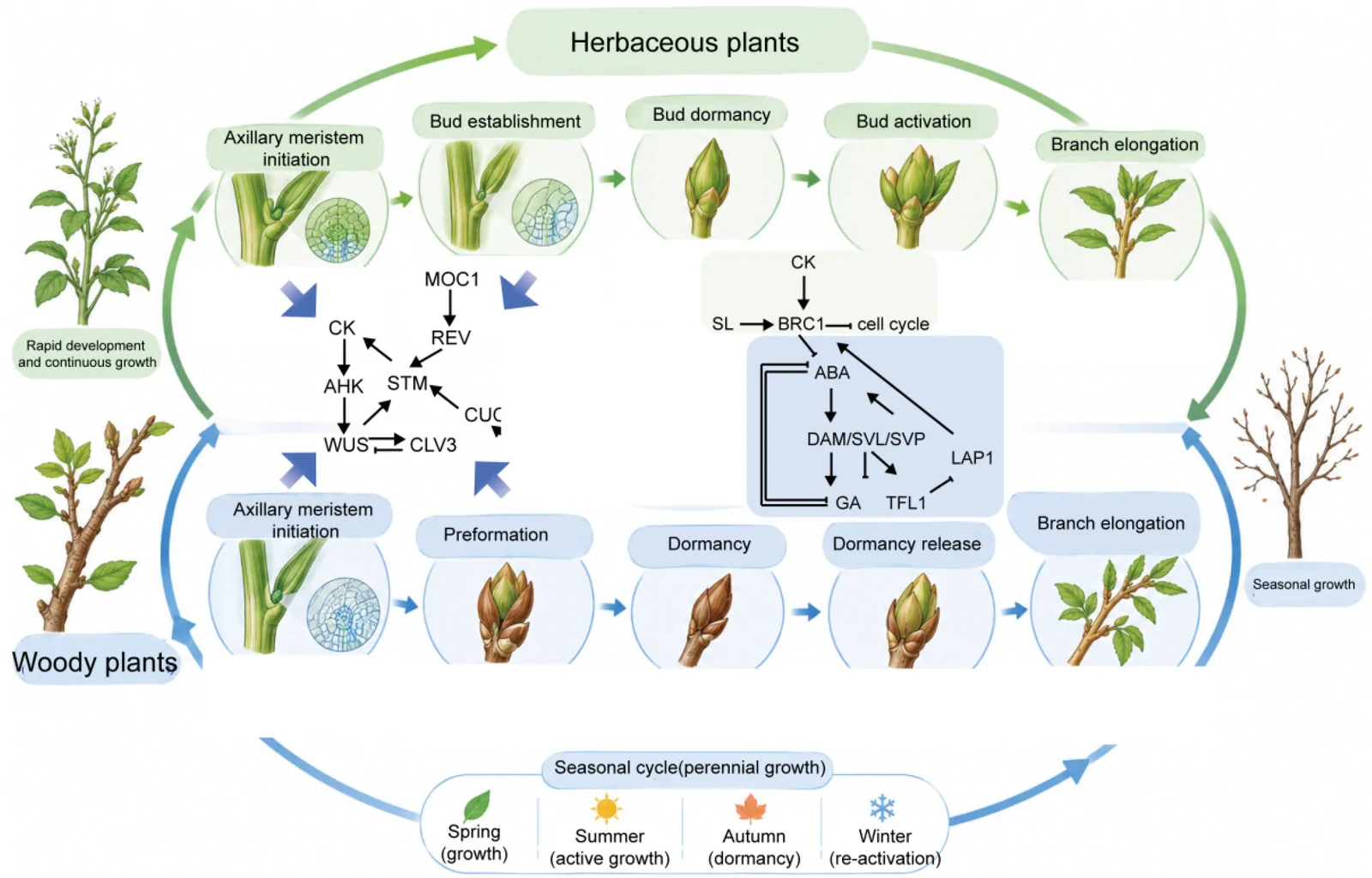
Developmental progression of shoot branching in herbaceous and woody plants. Note: Shoot branching follows a broadly conserved developmental sequence comprising axillary meristem initiation, bud establishment, dormancy, bud activation, and branch elongation, but these developmental transitions occur within distinct temporal and physiological contexts in herbaceous and woody plants. Green represents herbaceous plants, whereas blue represents woody plants. In herbaceous plants, shoot branching is generally integrated with relatively continuous vegetative growth, whereas woody perennials integrate bud preformation, seasonal dormancy, dormancy release, and subsequent branch elongation into recurrent growth cycles. Representative hormonal and transcriptional regulators are shown to highlight conserved regulatory modules and their integration with seasonal control of bud fate in woody plants.

Although the developmental framework of AM initiation appears to be broadly conserved across many studied seed plant species, notable differences exist between herbaceous and woody species [64]. In annual herbaceous plants such as Arabidopsis and rice, axillary meristems are established rapidly following leaf initiation and often proceed directly toward axillary bud development [11]. In contrast, perennial woody species undergo repeated cycles of vegetative growth over many years, during which AM formation is closely coordinated with seasonal environmental signals and perennial growth habits [65,66]. Homologs of *STM*, *LAS*, *RAX*, and other conserved regulators have been identified in poplar, apple, and additional tree species [67,68]. suggesting that conserved meristem regulatory modules are present in woody plants. Shoot branching can be viewed as a continuous developmental program comprising five interconnected stages (Figure 1). This framework represents an integrative model based on current knowledge rather than a universal mechanism established for all plant species. During AM initiation and bud establishment, meristematic competence is established in the leaf axil, followed by organ primordium formation and vascular connection with the main shoot. Bud dormancy or growth arrest is characterized by reduced cell division and organ growth while developmental potential is retained. Subsequent bud activation involves the reactivation of cell proliferation and organ growth, whereas sustained branch elongation depends on coordinated cell proliferation, expansion, differentiation, and vascular development. Although these developmental stages are frequently observed across both herbaceous and woody plants, their temporal organization and regulatory contexts differ (Figure 1 and Figure 2). In herbaceous plants, bud formation and outgrowth are often integrated with relatively continuous vegetative growth, whereas woody perennials frequently separate bud formation, growth arrest, and subsequent outgrowth across successive seasons. In woody plants, bud dormancy and activation are therefore closely associated with seasonal cues and changes in hormonal status, whereas growth arrest in herbaceous plants is more often determined by local developmental and physiological signals. By contrast, in annual herbaceous species, axillary bud growth is generally regulated by local developmental status, hormonal balance, and environmental conditions rather than by prolonged seasonal dormancy.

### 2.2. Axillary Bud Establishment

Following axillary meristem (AM) initiation, the newly specified meristem undergoes a progressive maturation process that culminates in the establishment of a functional axillary bud [8]. This developmental transition extends beyond the specification of stem cell identity and involves coordinated meristem organization, organogenesis, vascular differentiation, and physiological maturation [69]. As a consequence, the developing bud acquires the structural integrity and developmental competence required either to remain quiescent or to initiate branch growth in response to endogenous and environmental signals [70,71]. Therefore, axillary bud establishment represents a critical developmental checkpoint linking meristem initiation to subsequent bud fate determination [72].

A defining feature of bud establishment is the progressive maturation of the stem cell niche in *Arabidopsis thaliana* and *Nicotiana tabacum* [73,74,75,76]. Once an axillary meristem has been specified, its transition into a functional bud requires progressive expansion and maturation of the meristematic domain. Increasing *STM* activity has been shown to promote meristem proliferation and expansion, followed by activation of *WUS* and establishment of the canonical *CLV* feedback loop in model species such as Arabidopsis [67]. These regulatory relationships provide important insights into stem cell niche maintenance; however, the extent to which the same regulatory hierarchy operates in diverse herbaceous and woody species remains under investigation. This maturation process converts a newly specified meristem into a self-maintaining developmental unit capable of sustained organogenesis (Figure 2).

Concurrently, the developing meristem undergoes rapid morphogenesis. Successive initiation of leaf primordia generates the characteristic layered architecture of the axillary bud, while boundary tissues surrounding the bud maintain its physical separation from adjacent organs and preserve meristem identity [8,61,77]. The transition from a newly initiated meristem to an established bud is accompanied by extensive physiological reprogramming [78,79,80]. Cytokinin has been demonstrated to contribute to stem cell maintenance and meristem proliferation, whereas auxin exported from the developing bud has been implicated in vascular canalization and tissue patterning [81,82]. Additional regulatory pathways, including strigolactone, sugar signaling, gibberellin, and brassinosteroid pathways, have been suggested to influence bud maturation by coordinating cell division, vascular differentiation, and metabolic status [25,81]. Rather than acting independently, these signals are proposed to converge on interconnected transcriptional networks to synchronize cellular proliferation with tissue differentiation, thereby ensuring robust bud establishment before the developmental decision between dormancy and sustained growth is made [65,81].

Although the fundamental processes governing bud establishment appear to be conserved across many studied seed plant species, their developmental outcomes differ substantially between annual herbaceous and perennial woody species [83]. In annual species such as Arabidopsis and rice, axillary buds typically complete maturation rapidly and often proceed directly to outgrowth or enter only transient dormancy [47]. By contrast, in perennial woody plants, structurally mature buds frequently undergo prolonged seasonal dormancy before reactivation in subsequent growing seasons [65,83]. Thus, in woody species, successful bud establishment not only requires meristem maturation but also contributes to preparing buds for long-term maintenance and repeated cycles of dormancy and reactivation, thereby integrating branching with perennial growth strategies and seasonal environmental adaptation [65,66].

### 2.3. Bud Dormancy

Following bud establishment, axillary buds do not necessarily resume immediate growth. Instead, many buds enter a dormant state in which meristem viability is maintained while cell proliferation and organ growth are temporarily arrested [7,10]. Dormancy represents a reversible developmental program that allows buds to preserve their regenerative capacity until environmental conditions and endogenous physiological status become favorable for branch formation [66]. By temporally uncoupling bud formation from bud outgrowth, dormancy can provide plants with developmental flexibility to optimize branching architecture in response to changing environmental and physiological conditions [5]. In perennial species, this strategy is particularly important for synchronizing shoot development with seasonal environmental cycles, thereby contributing to winter survival and timely growth resumption under favorable conditions [5].

Bud dormancy is not a uniform physiological state but comprises several distinct regulatory phases [5]. According to the classical classification, dormancy can be divided into paradormancy, endodormancy, and ecodormancy, depending on whether growth inhibition is imposed by other plant organs, intrinsic properties of the bud itself, or unfavorable environmental conditions, respectively. In annual herbaceous species, paradormancy is commonly associated with apical dominance, whereas perennial woody plants typically undergo seasonal transitions from paradormancy to endodormancy and subsequently to ecodormancy before bud break [7,15]. The relative contribution and temporal sequence of these dormancy states, however, vary among species and developmental contexts.

At the cellular level, dormant buds remain metabolically active despite the absence of visible growth [84,85,86]. Cell division is markedly reduced, plasmodesmata become progressively blocked by callose deposition, and long-distance transport of photoassimilates, hormones, and signaling molecules is restricted, thereby isolating the dormant meristem from surrounding tissues [66]. Nevertheless, the stem cell niche remains intact, allowing buds to rapidly resume meristematic activity once dormancy is released [87]. This reversible maintenance of stem cell competence distinguishes dormancy from irreversible developmental arrest and provides the cellular basis for subsequent branch outgrowth.

The transition into and maintenance of bud dormancy are regulated through extensive interactions among phytohormones, environmental signals, and transcriptional networks [88,89]. Among these regulators, abscisic acid (ABA) has been widely implicated as an important regulator of dormancy establishment and maintenance [90], with particularly substantial evidence available from temperate perennial woody plants; for example, in peach and other temperate perennial woody species [91,92]. Environmental cues such as shortening photoperiods and decreasing temperatures can promote changes in ABA accumulation, which may contribute to the suppression of cell-cycle progression, restriction of symplastic connectivity, and enhancement of the expression of dormancy-associated genes. In perennial woody species, *DORMANCY-ASSOCIATED MADS-box* (*DAM*) [93] and *SHORT VEGETATIVE PHASE-LIKE* (*SVL*) transcription factors [88,94,95,96] have been implicated in integrating seasonal signals with ABA metabolism, forming regulatory modules that contribute to the establishment and maintenance of endodormancy [97,98,99,100] (Figure 2). By contrast, growth-promoting hormones, including cytokinins and gibberellins, generally accumulate during dormancy release and antagonize ABA signaling to restore meristem activity [101,102]. In addition, auxin and sugar signaling interact extensively with ABA-regulated pathways to coordinate dormancy progression and bud reactivation according to whole-plant developmental status and carbohydrate availability [103,104,105]. Together, these findings indicate that bud dormancy is governed by an integrated regulatory network rather than by a single hormonal pathway.

Although the molecular framework underlying bud dormancy has been intensively studied in herbaceous model species such as *Arabidopsis thaliana* and *Oryza sativa*, as well as in perennial woody species such as peach, apple, and poplar, increasing evidence suggests that similar regulatory principles also contribute to the maintenance of axillary bud quiescence in annual plants [7]. In annual herbaceous species such as Arabidopsis, *BRANCHED1* (*BRC1*) functioning as an important regulatory component rather than a universal “master regulator” of bud fate [33,106,107,108]. Consequently, bud dormancy should be regarded as a continuum of reversible growth repression whose duration and regulatory complexity vary among species according to life-history strategy, rather than as a process unique to perennial plants [66]. This developmental continuum establishes the physiological foundation for the subsequent transition from dormancy to bud activation (Figure 2).

### 2.4. Bud Activation

Bud activation represents the developmental transition from a dormant but developmentally competent axillary bud to a rapidly growing lateral shoot [15]. Unlike bud establishment, which generates a structurally complete meristem, bud activation involves the re-initiation of cell proliferation, restoration of metabolic activity, and establishment of sustained organ growth [77]. This transition is highly plastic and integrates endogenous developmental status with environmental and physiological signals, enabling plants to adjust branching patterns according to resource availability and external conditions [109]. Consequently, bud activation can be regarded as an important developmental checkpoint influencing shoot branching plasticity.

Increasing evidence indicates that bud activation is initiated by rapid metabolic reprogramming rather than by hormonal changes alone [104,110]. Following decapitation or an increase in carbohydrate availability, sucrose is rapidly redistributed to axillary buds, where it functions not only as a carbon source but also as an early signaling molecule [111]. Elevated sugar availability stimulates cellular metabolism, promotes sink strength, and activates trehalose-6-phosphate (Tre6P)-dependent signaling pathways, thereby establishing the metabolic competence required for renewed growth [112]. Notably, sugar accumulation precedes detectable changes in auxin transport, suggesting that carbohydrate signaling represents one of the earliest events initiating bud release [113].

Concomitant with metabolic activation, extensive hormonal reprogramming establishes a permissive environment for sustained bud growth [15]. In *Arabidopsis thaliana* and *Rosa hybrida*, Cytokinin promotes stem cell proliferation and the expression of cell cycle regulators, whereas strigolactones reinforce dormancy by suppressing bud growth potential [103,114]. In *Arabidopsis thaliana*, Apically derived auxin continues to exert indirect inhibition through modulation of cytokinin biosynthesis, strigolactone production, and auxin canalization rather than acting directly within the bud [115]. A central component of this regulatory network is *BRANCHED1*, which integrates hormonal and metabolic signals to determine the developmental competence of individual buds [106]. Downregulation of *BRC1* is generally associated with bud activation, although increasing evidence indicates that *BRC1*-independent pathways also contribute to bud release in several species.

Across diverse plant species, including *Arabidopsis thaliana*, *Oryza sativa*, and *Vitis vinifera*, Environmental signals profoundly influence bud activation by modulating both metabolic status and hormonal signaling [15]. Increased light intensity, favorable red to far-red light ratios, nutrient availability, and optimal temperatures generally promote bud outgrowth, whereas shade, drought, and nutrient limitation reinforce growth repression [116,117,118,119,120,121]. These environmental cues regulate bud activation through partially overlapping pathways involving carbohydrate allocation, cytokinin and strigolactone signaling, and, in many cases, modulation of *BRC1* expression [7]. Rather than functioning as independent inputs, environmental and endogenous signals form an interconnected regulatory network that allows buds to continuously assess developmental opportunities before committing to sustained branch growth.

Although the molecular mechanisms controlling bud activation are largely conserved among seed plants, their temporal dynamics differ substantially between annual herbaceous and perennial woody species [7,96]. In annual plants, bud activation often follows rapidly after release from apical dominance and contributes directly to canopy expansion and reproductive success [105]. In contrast, woody perennials typically activate axillary buds only after seasonal dormancy has been released, integrating photoperiod, temperature, and carbon availability with endogenous developmental programs [83]. Thus, bud activation represents a major developmental transition that converts a developmentally competent bud into an actively growing lateral shoot, linking environmental responsiveness to long-term shoot architectural plasticity (Figure 2).

### 2.5. Branch Elongation

Sustained branch elongation subsequently transforms the activated axillary bud into a functional lateral shoot capable of continuous extension and integration into whole-plant architecture [15]. The central challenge during branch elongation is to maintain meristem activity over an extended developmental period while coordinating cell proliferation, cell expansion, organogenesis, internode elongation, and vascular differentiation. These processes collectively determine the final length, thickness, spatial orientation, and longevity of lateral branches, thereby influencing canopy architecture, resource acquisition, and reproductive output [122,123]. Thus, sustained branch elongation can be viewed as a developmental process through which local growth is progressively translated into whole-branch architecture.

During this stage, hormonal regulation also shifts from controlling growth initiation toward maintaining tissue growth and coordinating branch architecture. In *Arabidopsis thaliana*, auxin synthesized continuously at the branch apex is transported polarly through the elongating stem, where it contributes to vascular differentiation, canalization of auxin transport, and maintenance of apical growth [115]. In *Arabidopsis thaliana* Gibberellins, Brassinosteroids and Strigolactones play important roles in internode elongation by promoting both cell proliferation and cell expansion, whereas Brassinosteroids enhance cell-wall extensibility and act together with growth-promoting signals such as gibberellins to facilitate longitudinal cell expansion [124,125,126,127]. In Arabidopsis and tobacco, Cytokinins continue to support meristem activity and organogenesis, while strigolactones contribute more broadly to branch architectural regulation, including growth rate, branch angle, and the growth potential of subsequent axillary buds [128,129,130]. Thus, during sustained elongation, hormonal networks no longer primarily determine whether an axillary bud is released, but instead maintain meristem function, coordinate growth rates among different tissues, and integrate local growth into a lateral branch with a defined spatial architecture.

Sustained branch growth must also remain matched to continuously changing metabolic demands. As branches produce new leaves, internodes, and vascular tissues, their requirements for carbohydrates and mineral nutrients progressively increase, causing developing branches to become strong carbon sinks [105,110]. Continued carbohydrate supply provides not only the energy and carbon skeletons required for cell proliferation and expansion, but also supports cell-wall biosynthesis, vascular maturation, and further construction of tissue architecture. Consequently, the rate and duration of branch elongation depend on dynamic matching between source–sink relationships and the demands of actively growing tissues. In contrast to the activation stage, during which carbohydrate availability primarily contributes to the initiation of bud growth, carbon allocation during sustained elongation serves as a long-term support system for the expanding growth tissues and their increasing metabolic demands.

**Figure 3:**
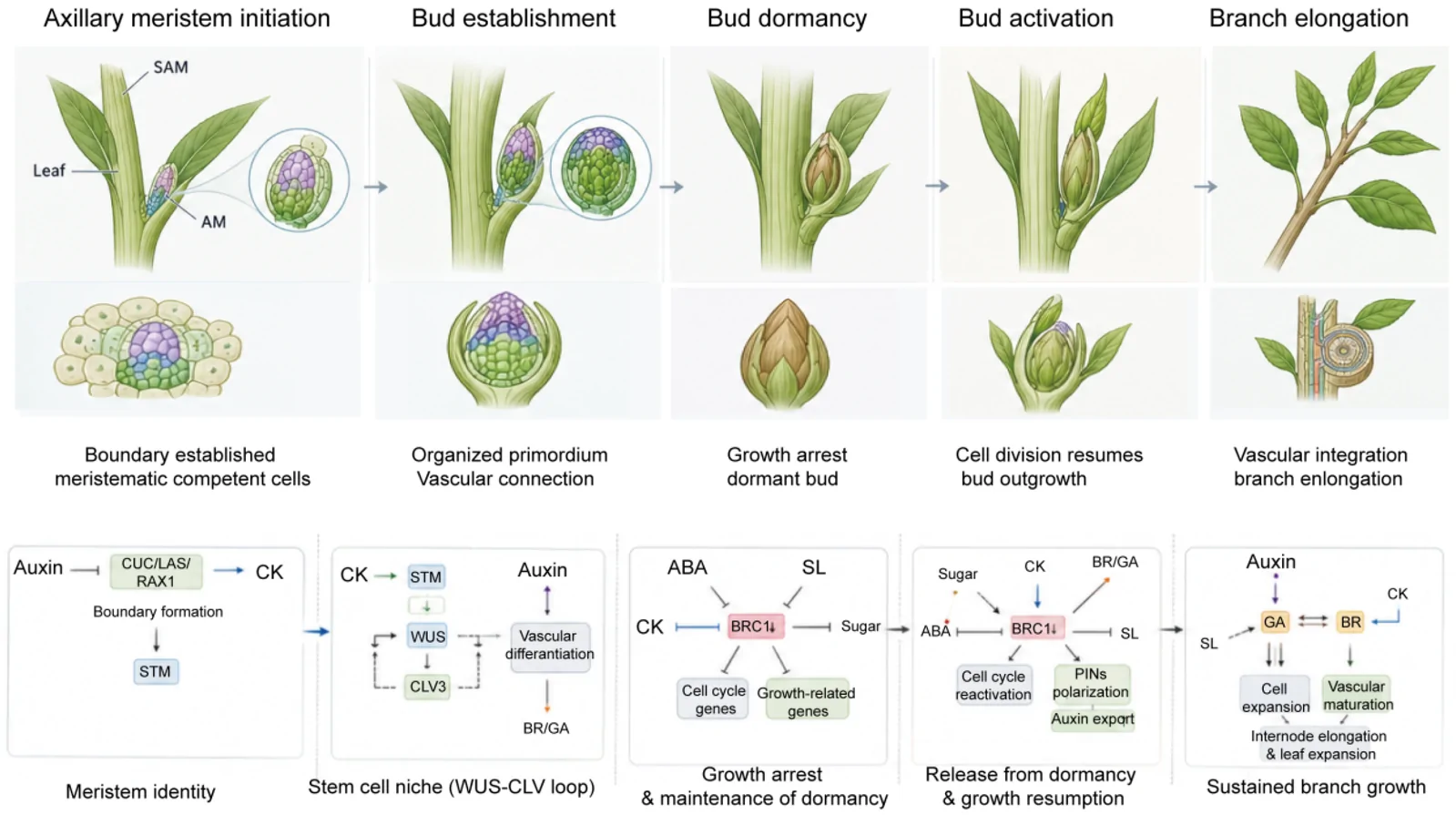
Regulatory dynamics of shoot branching in herbaceous and woody plants. Note: The upper panels compare representative morphological and cellular features of these developmental stages in herbaceous and woody plants, while the lower panels summarize the major hormonal and molecular regulatory modules involved at each stage. In the cellular schematics, purple indicates meristematic cells, including the shoot apical meristem (SAM) and axillary meristem (AM); green indicates developing bud tissues and leaf primordia; blue indicates vascular tissues or vascular connections; and pale yellow indicates surrounding and boundary cells.

Although the fundamental cellular and molecular mechanisms underlying sustained branch elongation are conserved in well-studied herbaceous models including Arabidopsis and rice, species differ substantially in their patterns of branch growth and architectural organization [15]. Environmental factors, including light quality, nutrient availability, water status, temperature, and biotic interactions, continuously modulate branch elongation and spatial orientation through coordinated effects on carbon allocation, hormonal homeostasis, meristem activity, and cellular growth [131]. In annual herbaceous plants, this process generally occurs within a relatively short life cycle and directly contributes to canopy expansion and reproductive structure formation. In perennial woody plants, by contrast, branch elongation is often integrated with seasonal growth, lignification, and the structural maintenance of perennial branches. Thus, conserved developmental modules can be differentially deployed across life-history contexts, generating substantial diversity in branch architecture (Figure 3).

## 3. Regulatory Networks Controlling Shoot Branching

The establishment of shoot architecture relies not only on the orderly progression of developmental events but also on the integration of multiple regulatory networks that determine the developmental fate of axillary buds [7]. From the specification of axillary meristems to branch elongation, each developmental transition is coordinated by dynamic interactions among phytohormones, transcriptional regulators, environmental signals, and cellular processes [15]. Rather than functioning as independent pathways, these regulatory modules operate as an interconnected signaling network that continuously integrates intrinsic developmental programs with external environmental information to optimize branching patterns according to developmental stage and resource availability [15,130].

Early studies of shoot branching primarily focused on the classical concept of apical dominance, in which auxin exported from the shoot apex suppresses the growth of axillary buds [132]. Subsequent discoveries of cytokinin—and strigolactone—mediated regulation substantially expanded this framework and established hormonal crosstalk as a central mechanism controlling branching decisions [82]. More recently, accumulating evidence has demonstrated that shoot branching is governed by a much broader regulatory landscape that includes sugar signaling, energy-sensing pathways, transcriptional regulatory modules, epigenetic regulation, and environmental cues such as light quality, nutrient availability, temperature, and water status [7]. These diverse regulatory pathways ultimately converge on a limited set of developmental regulators, including *STM*, *WUS*, and *BRC1*, which coordinate successive developmental transitions from meristem maintenance and bud establishment to dormancy, bud activation, and branch outgrowth [7,15,77].

**Figure 4:**
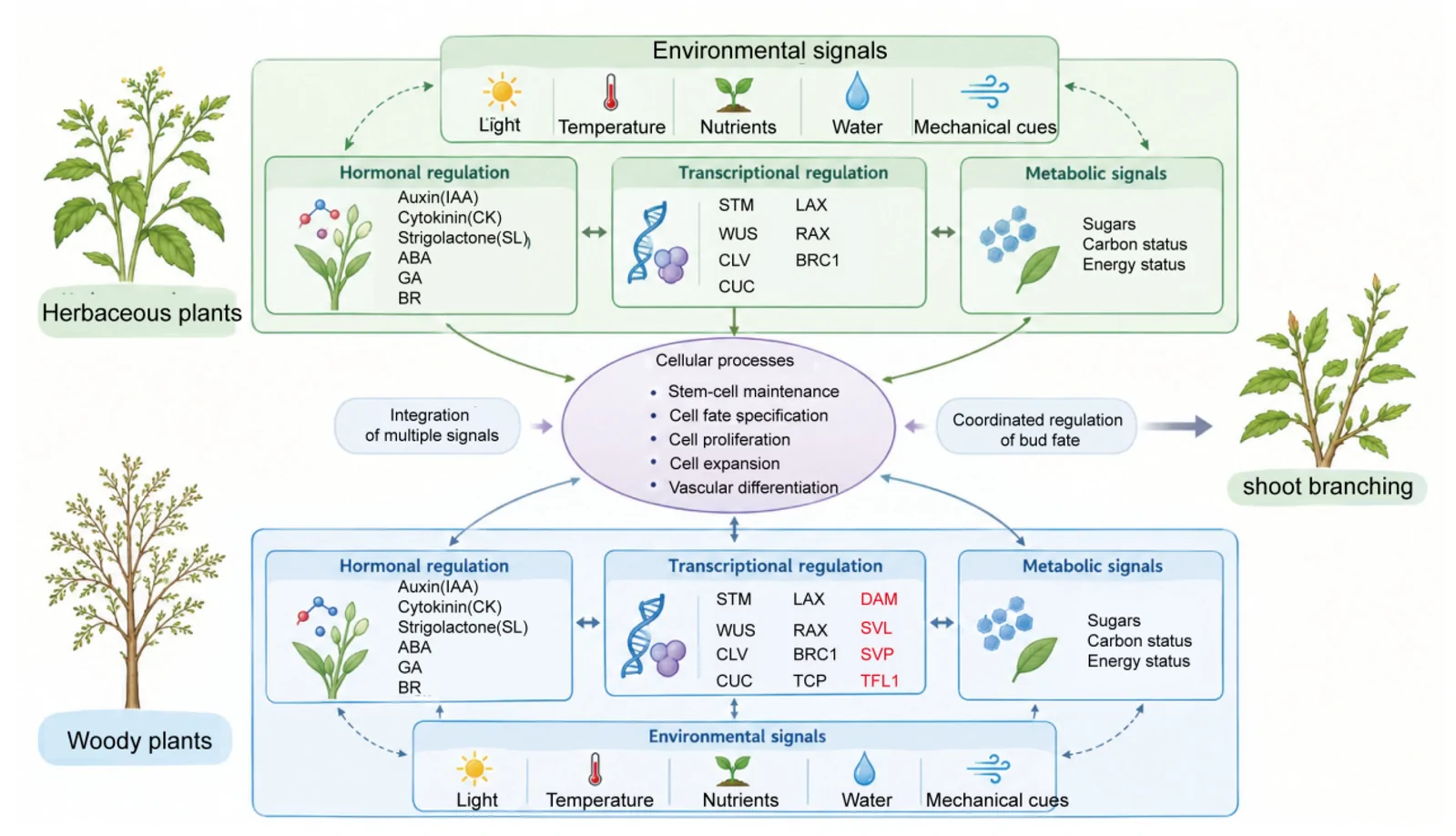
Integrated regulatory networks underlying shoot branching in herbaceous and woody plants. Note: Shoot branching is regulated by interactions among environmental signals (light, temperature, nutrient availability, water status, and mechanical cues), phytohormones, transcription factors, and metabolic signals (sugars, carbon status, and energy status). These regulatory inputs converge on cellular processes, including stem-cell maintenance, cell-fate specification, cell proliferation, cell expansion, and vascular differentiation, thereby coordinating bud development and shoot branching. The upper and lower panels illustrate representative regulatory frameworks in herbaceous and woody plants, respectively. Hormonal abbreviations: IAA, indole-3-acetic acid; CK, cytokinin; SL, strigolactone; ABA, abscisic acid; GA, gibberellin; BR, brassinosteroid. Transcriptional regulators include *STM, WUS, CLV, CUC, LAX, RAX, BRC1, TCP, DAM, SVL, SVP*, and *TFL1*. Arrows indicate regulatory relationships, and dashed arrows indicate proposed or context-dependent interactions. The model is schematic and may vary among species and developmental stages.

Importantly, the regulatory hierarchy controlling shoot branching is highly dynamic. Distinct signaling pathways predominate at different developmental stages [7]. During axillary meristem initiation, local auxin depletion and cytokinin accumulation cooperate with boundary-specific transcription factors to establish meristem identity [60,133]. As buds mature, transcriptional programs stabilize the stem-cell niche and promote organogenesis [134,135]. Following bud establishment, the balance among auxin, cytokinin, strigolactones, abscisic acid, and sugars determines whether buds remain dormant or resume growth, whereas continuous branch elongation depends on the coordinated regulation of cell-cycle progression, vascular development, and carbon allocation [7,15]. In this section, we summarize the major regulatory networks underlying shoot branching, focusing on four interconnected layers: hormonal regulation, transcriptional regulation, environmental regulation, and the cellular basis of shoot branching (Figure 4).

### 3.1. Hormonal Regulation

Phytohormones constitute the central regulatory framework governing shoot branching by coordinating developmental transitions from axillary meristem initiation to branch elongation [7]. Rather than functioning as independent regulators, hormones form a highly interconnected signaling network in which the spatial distribution, temporal dynamics, and reciprocal interactions of individual pathways collectively determine axillary bud fate. During the progression of shoot branching, the relative contribution of each hormone changes continuously, reflecting the distinct developmental requirements of meristem specification, bud maturation, dormancy maintenance, bud activation, and sustained branch growth.

Auxin occupies a central position within this regulatory hierarchy. Since the classical concept of apical dominance was established, auxin exported from the shoot apex has been recognized as a major systemic inhibitor of axillary bud outgrowth [82]. However, accumulating evidence indicates that auxin rarely suppresses bud growth directly. Instead, it regulates branching by modifying the hormonal environment surrounding the bud [136,137]. During axillary meristem initiation, a local auxin minimum within the leaf axil establishes a permissive environment for stem cell specification, whereas restoration of auxin transport following bud activation facilitates vascular canalization and sustained branch growth [7,115]. Thus, auxin exerts stage-dependent functions, promoting organ patterning while simultaneously restricting inappropriate bud activation through long-distance signaling [115].

In contrast to auxin, cytokinin primarily promotes shoot branching by enhancing meristematic activity and stimulating bud growth [60,82]. Local cytokinin accumulation within the leaf axil promotes the expression of stem-cell regulators such as *WUS* and supports the establishment of a functional stem-cell niche during axillary meristem formation [60,138,139]. Following bud establishment, cytokinin further promotes cell proliferation and facilitates bud activation, particularly under favorable environmental conditions [140]. Importantly, cytokinin activity is tightly coordinated with auxin signaling; auxin generally suppresses cytokinin biosynthesis in the stem, whereas localized cytokinin accumulation counteracts auxin-mediated inhibition within developing buds [7,82]. This antagonistic yet coordinated relationship enables plants to balance meristem maintenance with controlled branch production.

Strigolactones provide an additional regulatory layer by reinforcing bud inhibition and maintaining shoot architectural stability [141,142]. Acting downstream of auxin, strigolactones suppress bud activation through both transcriptional and non-transcriptional mechanisms [106,129,143]. Recent genetic and biochemical studies have established the D14–*MAX2–SMXL* signaling module as the core perception pathway, whereas the *TCP* transcription factor *BRC1*/*TB1* functions as a major downstream integrator that determines bud growth potential [106,144]. In addition to regulating transcriptional programs associated with bud dormancy, strigolactones reduce auxin export from activated buds, thereby limiting the establishment of stable polar auxin transport required for sustained branch elongation [115,143]. These findings support a revised view in which strigolactones primarily stabilize bud dormancy and restrict sustained outgrowth rather than simply preventing bud release.

Abscisic acid (ABA) predominantly functions during bud dormancy by maintaining growth arrest and preserving meristem viability under unfavorable developmental or environmental conditions [65,145]. In perennial woody species, the seasonal accumulation of ABA promotes endodormancy through interactions with dormancy-associated transcription factors and repression of cell-cycle activity [65,66]. Emerging evidence further indicates that *BRC1* can enhance ABA biosynthesis within axillary buds, establishing a positive regulatory module that reinforces dormancy maintenance [33]. Thus, ABA serves as a key hormonal link between environmental adaptation and developmental timing (Figure 2 and Figure 4).

### 3.2. Transcriptional Regulation

Unlike phytohormones, which often function as mobile signaling molecules, transcription factors establish cell-type-specific regulatory networks that determine meristem identity, boundary specification, bud fate, and sustained branch growth. Increasing evidence suggests that shoot branching is regulated by two major transcriptional hubs: one centered on the establishment and maintenance of meristematic competence and the other controlling the activation or repression of axillary bud growth.

In *Arabidopsis thaliana*, The initiation of axillary meristems depends on the establishment of a stable stem-cell niche [146]. Among the transcription factors involved, *STM* serves as the central regulator of meristem identity. Low but persistent *STM* expression in boundary cells preserves their meristematic competence before visible meristem formation, whereas subsequent upregulation of *STM* initiates axillary meristem development [146]. *STM* also promotes cytokinin biosynthesis through activation of *IPT* genes, thereby reinforcing meristematic activity and establishing a positive regulatory environment for stem-cell maintenance [147]. Following cytokinin accumulation, *WUS* is activated, and together with *CLV3* forms a conserved negative-feedback loop that stabilizes the newly established stem-cell niche [148]. This *STM*–CK–*WUS*–*CLV* module therefore represents the core transcriptional network underlying meristem establishment and maintenance.

The acquisition of meristem identity is spatially restricted to the leaf axil through the activity of boundary-specific transcription factors. Members of the *CUC* family establish the organ boundary and maintain a low-auxin microenvironment that preserves meristematic competence [54,149]. Downstream of *CUC* genes, *LAS* and *RAX* function cooperatively to activate *STM* expression and promote axillary meristem initiation [77,133,150]. Additional regulators, including *REV*, *DRN/DRNL*, *LOF*, further integrate positional information to ensure that axillary meristems are initiated only within appropriate boundary domains [151,152,153]. Rather than functioning in linear pathways, these transcription factors constitute an interconnected regulatory network that couples organ boundary formation with stem-cell specification.

Once axillary buds have formed, transcriptional regulation shifts from establishing meristem identity to determining bud fate. The *TCP*-family transcription factor *BRC1* has emerged as the principal integrator of this developmental transition. *BRC1* is preferentially expressed in axillary buds, where it suppresses bud outgrowth without affecting bud initiation [106]. Multiple upstream pathways converge on *BRC1*, including strigolactone, cytokinin, auxin, sugar signaling, and light responses, enabling individual buds to integrate endogenous and environmental information before committing to sustained growth [7]. High *BRC1* activity is generally associated with dormancy maintenance, whereas reduced *BRC1* expression accompanies bud activation [106]. However, accumulating evidence indicates that several signals can regulate axillary bud outgrowth through mechanisms that are at least partly independent of *BRC1*, including sugar/Tre6P signaling and strigolactone-associated regulation of auxin transport [106,154,155,156,157].

Beyond *BRC1*, several additional transcription factor families contribute to bud fate determination. Members of the *SPL*, *TCP*, *HD-ZIP III*, and *WOX* families regulate branching by interacting with hormonal pathways and developmental programs [7,130]. Importantly, related transcriptional regulators and their associations with shoot branching have also been identified in perennial woody plants, suggesting that key components of these regulatory modules are at least partly conserved across herbaceous and woody species, although their relative contributions and regulatory relationships may vary with species and developmental context [158,159]. Thus, transcriptional regulation extends beyond meristem specification and bud fate determination to directly control the cellular basis of branch development (Figure 4).

### 3.3. Environmental Regulation

Shoot branching exhibits remarkable developmental plasticity, enabling plants to dynamically adjust their architecture in response to fluctuating environmental conditions. Unlike intrinsic developmental programs, which define the potential for branch formation, environmental signals determine whether and when this developmental potential is realized. Light quality, temperature, water availability, mineral nutrients, and biotic interactions continuously modify branching patterns by influencing hormonal homeostasis, carbon allocation, and transcriptional regulatory networks [7,15,130].

In both Arabidopsis and chrysanthemum, light is one of the most influential environmental cues regulating shoot branching. Plants perceive changes in light intensity, photoperiod, and particularly the red-to-far-red (R:FR) ratio through phytochromes, allowing them to detect neighboring vegetation and initiate shade-avoidance responses [160,161,162,163]. Under low R:FR conditions, bud outgrowth is rapidly suppressed, reducing lateral branching while promoting elongation of the primary shoot [160]. This response is mediated through coordinated changes in auxin, cytokinin, and strigolactone signaling, together with increased expression of *BRC1* in axillary buds [7]. Light also affects carbon assimilation and sugar availability, providing an additional mechanism through which photosynthetic activity influences branching decisions [111]. Thus, light simultaneously regulates shoot branching through photoreceptor-mediated signaling and carbon metabolism.

In Arabidopsis, temperature and seasonal cues are particularly important for perennial woody species, where shoot branching must be synchronized with annual growth cycles. Short photoperiods and low temperatures promote bud dormancy and suppress bud outgrowth, whereas warming temperatures and favorable day lengths stimulate dormancy release and branch development [65,66,164]. Seasonal regulation involves extensive interactions among ABA, gibberellins, cytokinins, and dormancy-associated transcription factors, ensuring that branch growth occurs only under favorable environmental conditions [88]. In annual herbaceous species, temperature primarily influences branching by altering developmental rate, hormonal balance, and meristem activity rather than inducing prolonged seasonal dormancy [88]. These contrasting responses illustrate how conserved regulatory pathways have been adapted to distinct life-history strategies.

In Arabidopsis, nutrient availability provides another major environmental input controlling shoot architecture. Nitrogen and phosphate availability strongly influence branch number by regulating both hormone biosynthesis and carbon allocation [15,165,166]. Nutrient deficiency generally enhances strigolactone biosynthesis and, particularly under nitrogen limitation, reduces cytokinin accumulation, thereby reinforcing bud inhibition. Conversely, improved nutrient supply promotes cytokinin accumulation, increases sugar availability, and facilitates bud activation and sustained branch growth. Recent studies further demonstrate that nutrient responses converge on key regulatory nodes such as *BRC1*, integrating nutritional status with developmental programs [166]. These findings suggest that nutrient signaling does not act independently but is tightly coupled with hormonal and metabolic networks to optimize resource allocation between primary growth and branch production.

Water availability and other abiotic stresses further reshape shoot branching by modifying both hormonal signaling and metabolic activity. Drought commonly suppresses branch formation through increased ABA accumulation, reduced cytokinin biosynthesis, and decreased carbohydrate availability [167,168] (Figure 4).

### 3.4. Cellular Basis of Shoot Branching

Although hormonal and transcriptional networks determine the developmental competence of axillary buds, shoot branching is ultimately executed through coordinated cellular behaviors [7]. Every developmental transition, from axillary meristem initiation to sustained branch elongation, requires precise regulation of stem-cell maintenance, cell fate specification, cell proliferation, tissue differentiation, and intercellular communication [51,169,170,171]. Rather than acting independently, these cellular processes integrate diverse regulatory inputs to transform molecular signals into morphological development. Consequently, understanding the cellular basis of shoot branching provides the mechanistic link between gene regulatory networks and the establishment of plant architecture.

In *Arabidopsis thaliana*, the capacity to generate a new branch originates from a population of undifferentiated meristematic cells that retain stem-cell identity within the leaf axil [49,63]. During axillary meristem initiation, these cells are preserved in a slowly dividing state before becoming reactivated to establish a functional stem-cell niche [50]. Maintenance of this undifferentiated cell population primarily depends on sustained low-level *STM* expression, whereas the *WUS–CLV* regulatory circuit is established later to maintain stem-cell homeostasis in the newly formed axillary meristem [49]. Beyond transcriptional regulation, recent studies demonstrate that intercellular communication mediated by peptide signals, plasmodesmata, and mobile transcription factors is essential for maintaining meristem homeostasis. These mechanisms ensure that stem-cell populations remain stable while continuously supplying founder cells for organogenesis.

Following bud activation, sustained branch growth requires extensive cell proliferation, vascular differentiation, and cell expansion. In *Arabidopsis thaliana*, re-entry into the cell cycle during axillary bud activation and subsequent outgrowth is associated with activation of the conserved CYCD–CDKA–RBR–*E2F* regulatory pathway, which promotes G1-to-S phase progression and supports renewed cell proliferation [172]. Consistent with a role for CYCD3 in shoot branching, CYCD3-related mutants in Arabidopsis exhibit reduced axillary bud growth [173]. In rice, disruption of CYCD3 has also been associated with reduced tiller development, providing evidence that CYCD3-dependent cell-cycle regulation contributes to lateral shoot formation in grasses [174]. Importantly, in the woody tree Populus, overexpression of CYCD3 has been reported to promote cell proliferation and increase shoot branching [175], suggesting that CYCD3-mediated regulation of the cell cycle may also contribute to branch development in woody plants.

Successful branch establishment requires not only meristem activity but also the integration of the developing branch into the vascular network of the parental shoot. During bud activation, procambial cells differentiate into continuous xylem and phloem strands, establishing functional transport pathways for water, nutrients, sugars, hormones, and signaling molecules [7,176,177]. In Arabidopsis, polar auxin transport promotes vascular canalization, while coordinated cell-wall remodeling and plasmodesmatal connectivity facilitate communication between the developing branch and surrounding tissues [115,178]. Mechanical properties of the cell wall and extracellular matrix also influence cell expansion, tissue patterning, and organ stability, highlighting the importance of physical as well as biochemical regulation during branch development [179,180,181]. These cellular events collectively ensure that newly formed branches become physiologically integrated with the whole plant.

**Figure 5:**
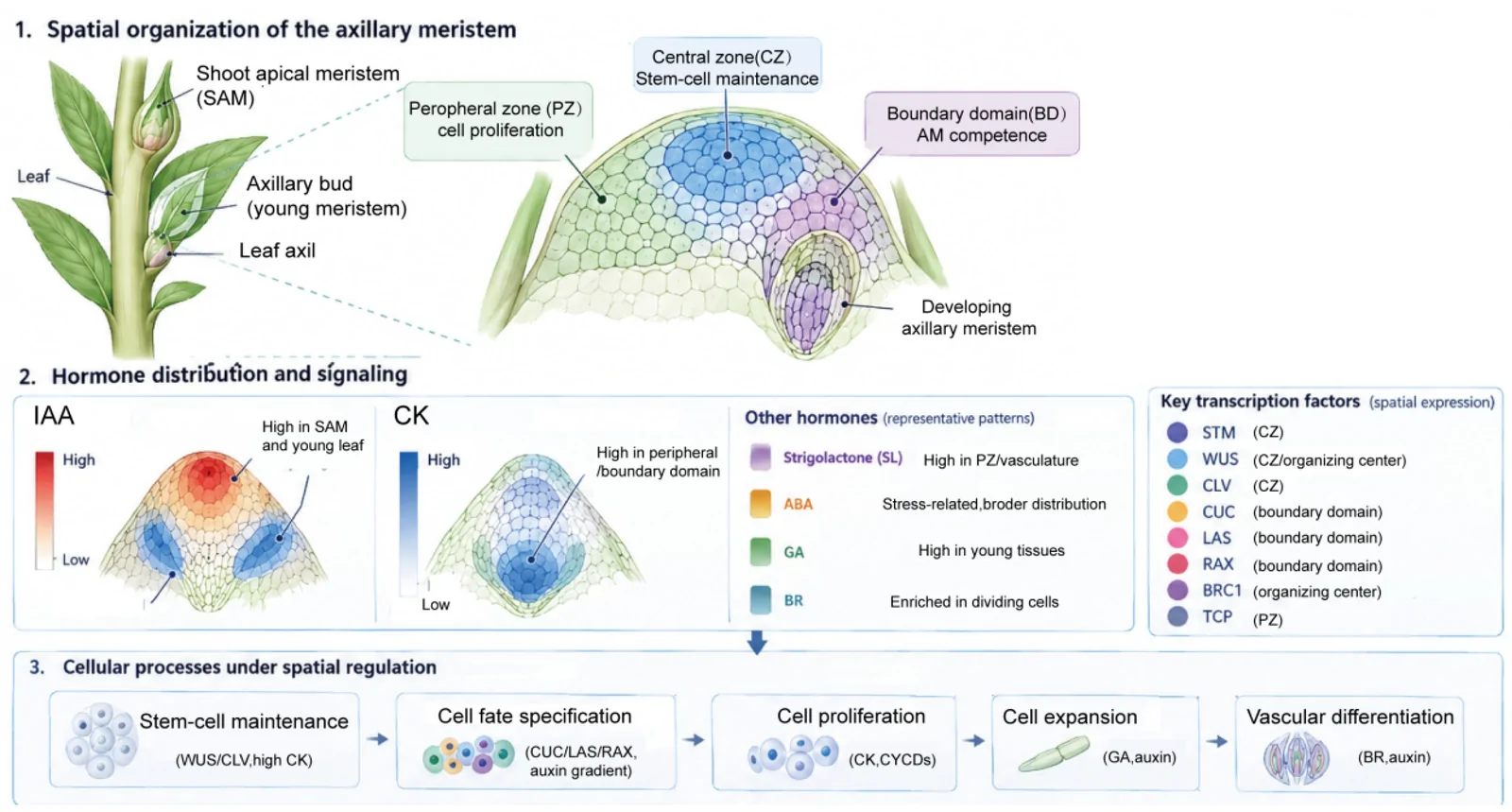
Spatial and cellular foundations of shoot branching Note: Spatial organization and hormonal regulation of the axillary meristem. (**1**) Spatial organization of the axillary meristem (AM) in the leaf axil, adjacent to the shoot apical meristem (SAM). The central zone (CZ) is associated with stem-cell maintenance, the peripheral zone (PZ) with cell proliferation, and the boundary domain (BD) with AM competence. (**2**) Representative spatial distributions of phytohormones and the expression domains of key transcriptional regulators. IAA, indole-3-acetic acid (auxin); CK, cytokinin; SL, strigolactone; ABA, abscisic acid; GA, gibberellin; BR, brassinosteroid. The color gradients indicate relative hormone abundance or signaling activity, with red and blue gradients representing IAA and CK, respectively; the other hormone colors denote representative distribution patterns rather than quantitative measurements. (**3**) Major cellular processes associated with AM development, including stem-cell maintenance, cell-fate specification, cell proliferation, cell expansion, and vascular differentiation. Key transcriptional regulators are shown according to their representative spatial expression domains: *STM*, *WUS*, *CLV*, *CUC*, *LAS*, *RAX*, *BRC1*, and *TCP* family transcription factors. The hormone abbreviations in parentheses below the cellular processes indicate representative hormonal associations, not necessarily exclusive regulatory inputs. Arrows indicate the progression or conceptual relationship among the illustrated developmental processes. The spatial patterns and regulatory relationships are schematic and may vary among species and developmental stages.

Shoot branching is regulated within a highly organized spatial cellular framework, in which different regions of the axillary meristem exhibit distinct developmental potentials, hormonal activities, and transcriptional states (Figure 5). The central zone (CZ), peripheral zone (PZ), and boundary domain (BD) provide distinct cellular contexts for stem-cell maintenance, cell proliferation, and axillary meristem competence, respectively. The developing axillary meristem emerges near the leaf–stem boundary, indicating that cellular developmental potential depends not only on intrinsic molecular states but also on spatial position.

Hormonal signals and transcriptional programs are also spatially regulated. Auxin and cytokinin show distinct distribution patterns within the meristem and surrounding tissues, and their relative activities contribute to axillary meristem establishment and maintenance. For example, a local auxin minimum in the Arabidopsis leaf axil precedes an increase in cytokinin signaling. Meanwhile, *STM*, *WUS*, *CUC*, *LAS*, and *RAX* show spatially restricted expression patterns that collectively regulate meristem identity, boundary formation, and cellular proliferation and differentiation. Thus, spatial hormone distributions and gene-expression patterns contribute to defining the developmental competence of distinct cellular domains. These spatially defined molecular states ultimately converge on cellular processes, including cell-fate specification, cell proliferation, cell expansion, and vascular differentiation, which drive axillary meristem formation, bud development, and subsequent branch elongation. Shoot branching can therefore be viewed as a continuous developmental process in which spatial information, hormonal activity, and transcriptional states define cellular competence, while coordinated cellular behaviors translate this competence into organ formation and growth.

## 4. Evolution and Applications

The remarkable diversity of shoot architectures observed across land plants reflects the evolutionary diversification of regulatory mechanisms controlling shoot branching. Although the fundamental developmental framework of axillary meristem initiation, bud dormancy, and branch outgrowth is broadly conserved, the molecular networks governing these processes have undergone extensive evolutionary modification during the transition from early land plants to modern angiosperms [7]. Gene duplication, functional divergence, and the rewiring of regulatory networks have collectively enabled plants to generate species-specific branching patterns that maximize fitness under diverse ecological conditions [182,183]. Consequently, understanding the evolutionary trajectory of shoot branching provides not only insight into the origin of plant architectural diversity but also valuable resources for crop improvement and forest tree breeding.

Comparative genomic and evolutionary studies indicate that several core developmental regulators, including class I KNOX (*STM*), NAC-domain boundary genes (*CUC*), and *PIN*-mediated auxin transport, originated early during land plant evolution and have remained broadly conserved across major land plant lineages, whereas the stem-cell regulatory role of *WUS* evolved later within vascular plants [184]. These conserved regulators establish the fundamental developmental machinery required for meristem maintenance and branch formation [7]. In contrast, downstream regulatory modules controlling bud fate, hormone sensitivity, and environmental responsiveness have diversified substantially among evolutionary lineages. For example, the *TB1/BRC1* transcriptional module has undergone lineage-specific functional diversification and has become a major determinant of branching architecture in flowering plants [185], whereas perennial woody species have evolved additional regulatory circuits associated with seasonal dormancy, secondary growth, and long-term meristem maintenance [186]. These innovations illustrate how conserved developmental programs have been modified to accommodate contrasting life-history strategies and environmental adaptations.

The evolution of shoot branching has also been shaped by interactions between developmental regulation and environmental adaptation [9]. Annual herbaceous species generally favor rapid branch initiation and reproductive plasticity, whereas perennial woody plants coordinate branching with seasonal growth cycles, resource storage, and secondary vascular development [186]. Similarly, domestication has profoundly altered branching architecture in many crop species through artificial selection of key regulatory genes. Classical examples include *TB1* in maize, which contributed to the transition from highly branched teosinte to modern maize with reduced tillering, and *IPA1/SPL14* in rice, which optimized tiller number while improving grain yield [187,188]. Parallel domestication events affecting branching regulators have also been reported in wheat, barley, tomato, cotton, soybean, and numerous horticultural crops, demonstrating that shoot branching represents one of the most intensively selected developmental traits during crop evolution.

Recent advances in genomics and genome editing have greatly accelerated the translation of branching biology into practical breeding strategies. High-quality reference genomes, pan-genome analyses, genome-wide association studies (GWAS), quantitative trait locus (QTL) mapping, and comparative transcriptomics have facilitated the identification of branching-associated genes across diverse plant species [189,190]. Meanwhile, CRISPR/Cas-based genome editing has enabled precise modification of regulatory genes controlling branch number, branch angle, and tiller architecture without introducing undesirable pleiotropic effects. Rather than targeting single genes alone, current breeding strategies increasingly focus on fine-tuning regulatory networks by modifying promoter activity, cis-regulatory elements, or hormone sensitivity to achieve optimal plant architecture under specific production environments.

Beyond annual crops, understanding shoot branching is becoming increasingly important for perennial woody plants and forest tree improvement. Branch architecture directly influences canopy structure, timber quality, carbon sequestration, biomass production, and resistance to environmental stress [191]. Compared with annual species, woody plants exhibit unique developmental characteristics, including prolonged juvenile phases, seasonal bud dormancy, repeated cycles of cambial activity, and extensive secondary growth, indicating that additional regulatory mechanisms remain to be elucidated [186]. Integrating knowledge derived from model plants with emerging genomic resources in forest trees will therefore be essential for improving tree architecture, enhancing wood production, and increasing ecosystem resilience under future climate change.

## 5. Perspectives

**Figure 6:**
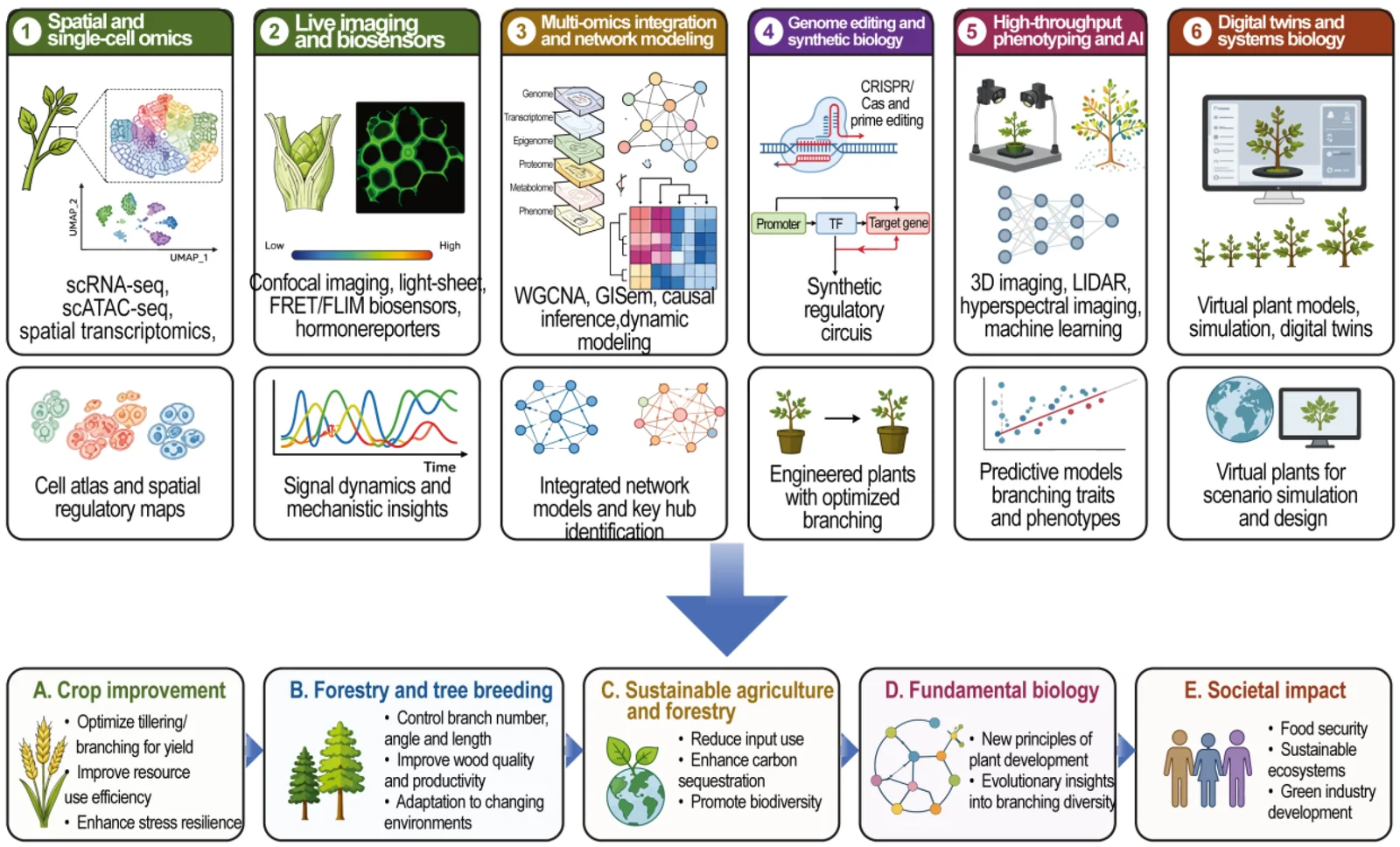
Emerging technologies and future perspectives for deciphering and engineering shoot branching. Note: Advances in spatial and single-cell omics, live imaging, multi-omics integration, genome editing, high-throughput phenotyping, and AI are transforming the study of shoot branching. Integrating these approaches enables the construction of predictive regulatory networks, precise manipulation of branching traits, and systems-level understanding of branch development.

Over the past decades, our understanding of shoot branching has evolved from descriptive developmental observations to an integrated framework encompassing hormonal signaling, transcriptional regulation, environmental responses, and cellular processes [7]. Nevertheless, a major challenge is to understand how these regulatory layers are coordinated across different cell types, developmental stages, and environmental conditions. This challenge is particularly evident in perennial woody plants, where seasonal dormancy, recurrent bud activation, secondary growth, and long-term meristem maintenance introduce additional temporal and spatial complexity [186]. Emerging technologies are now providing complementary approaches to address these questions at multiple biological scales (Figure 6).

Single-cell and spatial transcriptomics, live-cell imaging, quantitative hormone analysis, and multi-omics integration provide new opportunities to resolve the cellular and molecular mechanisms underlying shoot branching. For example, single-cell transcriptomics has been used to characterize cellular states in the Arabidopsis shoot apex and lateral organs, whereas spatial transcriptomics has been applied in maize and other species to resolve spatially restricted gene expression patterns in meristems, boundary domains, and developing organs [192,193]. However, single-cell approaches may disrupt native spatial relationships during tissue dissociation, whereas spatial transcriptomics remains constrained by spatial resolution and transcript capture efficiency. Moreover, changes in transcript abundance do not necessarily reflect protein activity or hormone status. Candidate regulatory modules identified in different species therefore require validation through live imaging, hormone measurements, and genetic approaches to distinguish correlation from causation.

Genome editing provides a direct approach for testing the causal roles of genes involved in shoot branching. CRISPR/Cas technologies have been applied to investigate shoot architecture in Arabidopsis, rice, maize, tomato, and other plants. For example, editing the strigolactone biosynthetic gene *CCD7* in rice increases tiller number but also reduces plant height [194], whereas modification of the strigolactone signaling and biosynthetic genes *SlD14* and *SlMAX1* in tomato alters axillary shoot growth but can also be accompanied by reduced yield and delayed fruit ripening [195]. These studies demonstrate the utility of genome editing for testing gene function and modifying plant architecture, but also highlight potential pleiotropic and unintended effects. Genetic background, editing strength, tissue specificity, developmental stage, and environmental conditions can influence the resulting phenotype, limiting direct extrapolation of regulatory effects across species.

High-throughput imaging, LiDAR, and hyperspectral phenotyping enable quantitative assessment of complex architectural traits, including branch number, angle, length, and spatial distribution, and are increasingly being applied to crops, horticultural plants, and woody species [196]. Three-dimensional phenotyping approaches such as LiDAR are particularly useful for characterizing complex canopy and branch structures, providing new opportunities for quantitative analysis of plant architecture. These technologies therefore currently serve primarily to improve the efficiency and scale of architectural phenotyping, while their predictive performance requires further validation across genetic backgrounds and environmental conditions.

Integrating molecular datasets, quantitative phenotypes, and computational models may further facilitate the prediction of shoot branching dynamics. Functional-structural plant models and virtual plant models can already simulate aspects of plant growth and spatial architecture under defined conditions and are increasingly being considered within digital-twin frameworks [197,198]. Recent plant digital-twin frameworks have further explored the integration of genomic, phenotypic, and environmental data to simulate plant growth and environmental responses. However, the application of digital twins to plant and agricultural systems remains under development and is constrained by challenges in data integration, model parameterization, interoperability, and large-scale validation. Digital twins should therefore currently be regarded primarily as tools for data integration, dynamic simulation, and hypothesis generation rather than mature systems capable of accurately predicting the complete architecture of individual plants.

Overall, single-cell and spatial omics, genome editing, high-throughput phenotyping, and computational modeling are progressively connecting molecular mechanisms, cellular behaviors, and whole-plant architecture, but their applications remain strongly dependent on species and environmental context. Future integration of multi-scale omics, live imaging, genome editing, quantitative phenotyping, and computational modeling may help establish causal relationships in shoot branching and determine the context dependence and potential pleiotropic effects of specific regulatory nodes across species and environments. Thus, shoot branching research may gradually move from mechanistic understanding toward more quantitative prediction and targeted manipulation, although precise “molecular design” of plant architecture will require further experimental validation and technological development.

## Data Availability

No new data were created or analyzed in this study. Data sharing is not applicable.
